# Influence of physical activity on perceived stress and mental health in university students: a systematic review

**DOI:** 10.3389/fspor.2025.1710832

**Published:** 2026-01-09

**Authors:** Alexandra Martín-Rodríguez, Natalia González-Prieto

**Affiliations:** 1 Faculty of Health Sciences, UNIE University, Madrid, Spain; 2 Faculty of Medicine, Health and Sports, Universidad Europea de Madrid, Madrid, Spain; 3 Faculty of Medicine, University of Málaga, Málaga, Spain

**Keywords:** active lifestyle, COVID-19 lockdown, emotional wellbeing, mental health outcomes, physical inactivity, psychological distress, sedentary behavior, university population

## Abstract

University students are a population particularly vulnerable to stress, anxiety, and reduced wellbeing. Physical activity has been proposed as a protective factor, but existing findings are heterogeneous. This systematic review examined the relationship between physical activity and mental health in university students, focusing on perceived stress, anxiety, depression, and psychological wellbeing. It was conducted in accordance with PRISMA guidelines, and the study protocol was registered in PROSPERO (CRD420251179614). A total of 38 studies published between 2020 and 2025 were analyzed, involving more than 20,000 participants from various countries. Most studies were cross-sectional, although some longitudinal and quasi-experimental studies were also included. The results showed a consistent association between higher levels of physical activity and lower levels of stress, depression, and anxiety, as well as an increase in subjective wellbeing. In addition, mediators such as sleep quality and resilience, and moderators such as gender or internet use, were identified. The effects were more significant when physical activity was combined with other healthy habits such as good sleep and low sedentary behavior. Although most of the studies were not experimental, the evidence suggested a possible beneficial causal effect of exercise. The need for comprehensive interventions in universities was highlighted, promoting physical activity as a preventive and therapeutic strategy to improve the mental health of students.

**Systematic Review Registration**: https://www.crd.york.ac.uk/PROSPERO/view/CRD420251179614, PROSPERO, CRD420251179614.

## 1 Introduction

The university stage coincides with a vital period of high perceived stress and mental health vulnerability among young adults. Various global reports show that university students experience worrying levels of stress and psychological distress. For example, according to a national college health survey in the United States, three out of four students reported feeling stressed, and nearly one in five admitted to experiencing stress-related suicidal ideation. This crisis worsened during the COVID-19 pandemic. In the 2020–2021 academic year, more than 60% of students met the criteria for at least one mental health issue, and 83% reported that psychological difficulties negatively affected their academic performance (1). These data highlight the magnitude of the problem and its practical implications, as sustained academic stress is linked to poorer educational performance and can be a predictor of future mental disorders. In fact, many conditions such as depression and anxiety often first emerge during the university years (2).

At the same time, sedentary behavior and physical inactivity have become increasingly common among university students, increasing both physical and psychological risks (3). Theoretical and empirical evidence suggests that regular physical activity confers significant mental health benefits. It helps regulate the stress response, improves mood, and enhances psychological wellbeing through physiological mechanisms (e.g., regulation of the stress-related neuroendocrine axis and endorphin release) and psychosocial mechanisms (e.g., increased self-esteem and social interaction) (4).

With regard to perceived stress, defined as the level of stress experienced according to an individual's subjective appraisal, numerous studies have found that physically active university students tend to report lower stress levels than their sedentary peers (5). For instance, staying active and reducing sedentary behavior are associated with significantly lower levels of perceived stress in this population. In addition, physical exercise has been linked to a lower incidence of depressive and anxiety symptoms, as well as better self-esteem and sleep quality, all factors related to improved overall mental health (6). In recent years, scientific interest in the influence of physical activity on the mental wellbeing of students has grown. Several studies and reviews support the beneficial effects of exercise on stress and psychological health in this population. For example, a recent systematic review identified a consistent association between higher physical activity and reduced stress among university students (3). Several empirical studies conducted during and after the COVID-19 pandemic highlight a significant reduction in physical activity among university students alongside increased psychological discomfort. In fact, evidence from Italian universities, one of the most affected academic contexts in Europe, reported marked declines in sport participation and negative effects on wellbeing (7, 8). Conversely, maintaining active habits during lockdowns was associated with lower psychological distress, particularly among women students (9). These findings emphasize the importance of physical activity in buffering stress during critical situations and underline cultural and contextual factors shaping the experiences of students.

Likewise, controlled exercise-based interventions have been shown to significantly reduce symptoms of anxiety, depression, and stress among students. A meta-analysis found that physical activity interventions in student populations reduced perceived stress with a moderate effect size (SMD = −0.61), along with similar or even greater effects on anxiety and depression symptoms. These findings underscore the theoretical importance of physical activity as a strategy for stress management and mental health promotion (3).

Previous studies have examined the associations between physical activity and mental health in young adults or general populations; however, most available research either covers broad age ranges, relies on mixed samples (combining adolescents and adults), or is limited to prepandemic data. Moreover, the literature has often concentrated on narrow psychological outcomes—typically depression or anxiety—while overlooking other relevant indicators such as perceived stress, wellbeing, or life satisfaction (3, 10, 11, 12). López-Valenciano et al., for instance, reported that decreases in physical activity during the pandemic were associated with greater psychological distress and lower wellbeing in university students. However, their review included studies conducted both before and during the early stages of COVID-19 and did not examine potential mediating factors such as sleep quality or sedentary behavior (10). Similarly, most analyses did not explore potential mediators or moderators such as sleep, sedentary behavior, or resilience. Therefore, despite existing evidence, a recent and more targeted synthesis focusing specifically on university students is needed to clarify how physical activity relates to perceived stress and mental wellbeing in this population.

In summary, understanding how physical activity influences perceived stress and other mental health indicators in university students is of both scientific and practical interest. Theoretically, it offers insight into stress-coping mechanisms and the modulatory role of physical exercise on young adult psychology. Practically, the results can inform university programs promoting healthy lifestyles, with physical activity as a key component for reducing academic stress and preventing emotional problems during a critical stage of development.

The aim of this systematic review is to comprehensively synthesize the current scientific evidence regarding the influence of physical activity on both perceived stress levels and overall mental health in university students. This review responds to the growing need to understand how lifestyle factors, particularly physical activity, can serve as protective or therapeutic mechanisms against the psychological challenges faced by students in higher education. More specifically, it seeks to

Assess the association between physical activity—whether measured as a habitual behavioral pattern (e.g., weekly frequency, intensity, and duration of exercise) or through structured exercise interventions (e.g., training programs and group fitness sessions)—and the levels of perceived stress reported by students enrolled in university or college programs. This includes examining to what extent physical activity contributes to stress reduction in different academic and cultural contexts, and whether specific exercise modalities or intensities are more effective.
Examine the impact of physical activity on various mental health outcomes, including but not limited to symptoms of anxiety, depression, academic distress, emotional wellbeing, life satisfaction, and overall psychological functioning. The review aims to explore both the mitigating effects of exercise on negative mental health indicators and its potential to promote positive states such as resilience, motivation, and self-esteem.

In addition, this review endeavors to integrate and critically analyze findings that are currently dispersed across diverse disciplines and methodologies, aiming to identify consistent trends, theoretical contributions, and empirical gaps in the literature. By doing so, it hopes to provide a solid foundation for the development of evidence-based and context-sensitive interventions that promote physical activity as a key component in enhancing the psychological wellbeing of students. Ultimately, this work aspires to support universities, health professionals, and policymakers in designing comprehensive wellness strategies that are preventive, accessible, and sustainable in the face of rising mental health concerns in academic environments.

## 2 Materials and methods

### 2.1 Design and guidelines

This study was conducted as a systematic review in accordance with PRISMA guidelines (Preferred Reporting Items for Systematic Reviews and Meta-Analyses), using a narrative synthesis approach due to heterogeneity in study designs and outcome measures (11). The study protocol was registered in PROSPERO (CRD420251179614). A systematic literature review was conducted also in accordance with the PRISMA guidelines to ensure a transparent and rigorous process. The research question was structured using the PICO model, with the following components:

Population (P): Undergraduate or postgraduate university students (young adults 18–30 years old), of any gender, enrolled in institutions of higher education were included. Studies focused exclusively on secondary school adolescents, the general non-university population, or other age groups were excluded, unless data for university students were reported separately.
Intervention/Exposure (I): Physical activity, broadly defined as the practice of physical exercise or sport, was analyzed. This included both habitual activity levels (e.g., min/week and compliance with WHO recommendations) and structured interventions (e.g., exercise programs, physical education classes) aimed at increasing physical activity. Studies focusing solely on other interventions (e.g., meditation and psychological therapy) without including physical exercise, or those measuring only physical fitness without linking it to psychological variables, were excluded.
Comparison (C): In observational studies, comparison groups were defined as students with low or no physical activity (i.e., sedentary lifestyle) vs. physically active students. In experimental studies (clinical trials, quasi-experiments), control groups with no intervention were included (e.g., students maintaining usual habits or receiving a non-exercise-related intervention).
Outcomes (O): Outcomes included perceived stress and mental health, measured using validated quantitative instruments. Perceived stress was preferably assessed with standardized scales (e.g., Cohen's Perceived Stress Scale—PSS) or academic stress scales. Mental health indicators encompassed both negative psychological symptoms (e.g., academic stress, anxiety, depression, and distress) and positive indicators (e.g., subjective wellbeing, life satisfaction, and resilience), evaluated through validated questionnaires, clinical diagnoses, or self-reports. Studies measuring only physical performance or isolated physiological markers (e.g., cortisol levels) without psychological variables, as well as those focusing solely on academic performance without measuring stress or wellbeing, were excluded.

### 2.2 Information sources and search strategy

Systematic searches were conducted in major international databases relevant to health sciences, psychology, and education: PubMed (Medline), Web of Science (WOS), and Scopus. In addition, regional databases and literature in Spanish (e.g., Dialnet) were consulted, along with Google Scholar, to include gray literature. The final Boolean search string used in all databases was as follows: “university students” OR “college students” OR undergraduates) AND (“physical activity” OR exercise OR sport OR “active lifestyle” OR fitness) AND (stress OR anxiety OR depression OR “mental health” OR wellbeing OR “psychological distress”).

 Because of the large number of results retrieved, focus was placed on the most recent studies. Thus, only studies published from 2020 onward were included, covering a 5-year research window. This time frame was selected because recent literature shows significant changes in physical activity patterns and mental health among university students during and after the COVID-19 pandemic, leading to a rapid increase in new research. Restricting the search to this period ensured that the review captured updated and postpandemic evidence. The search combined English terms related to physical activity (e.g., physical activity and exercise, sedentary) and mental health (e.g., stress, anxiety, depression, wellbeing, and mental health), using filters for university population (college OR university students).

Two reviewers independently screened titles, abstracts, and full texts, and interrater reliability was assessed using Cohen's kappa, which showed substantial agreement. Observational studies were evaluated using the Newcastle–Ottawa Scale (NOS) and experimental or quasi-experimental studies using the Cochrane Risk of Bias 2.0 (RoB 2) tool. ROBINS-I was applied when appropriate for non-randomized intervention studies. Studies rated as low quality or presenting high risk of bias were excluded from the final synthesis.

A language filter was applied to include studies published in English or Spanish, ensuring coverage of both global and regional scientific production. Initially, no restrictions were placed on study type to maximize sensitivity; inclusion/exclusion criteria were applied during later stages. Reference lists of included articles and previous reviews were also manually screened to identify additional eligible studies not captured in the electronic search. All references were managed using Mendeley reference software, and duplicates were removed before screening.

### 2.3 Inclusion and exclusion criteria

Inclusion and exclusion criteria were defined in accordance with the PICO framework:

Participants: Studies with samples composed primarily of undergraduate or postgraduate university students were included. Studies involving preuniversity adolescents, non-university general population, or unrelated groups were excluded.
Intervention/exposure: Studies that assessed physical activity as an independent or exposure variable were selected. These included both observational studies (e.g., associations between self-reported physical activity and mental health) and experimental/quasi-experimental studies (e.g., controlled trials and exercise interventions). Studies focusing exclusively on non-physical interventions (e.g., psychological therapy and yoga/meditation without exercise) or those assessing only physical fitness without psychological outcomes were excluded.
Outcomes: Studies had to report at least one measure of perceived stress and/or mental health (e.g., anxiety, depression, and wellbeing) in students. Accepted outcomes included general stress scores (e.g., PSS-10), academic stress, negative symptoms (e.g., anxiety, depression, and psychological distress), and positive indicators (e.g., subjective wellbeing, life satisfaction, and resilience). Studies evaluating only physical performance, purely physiological variables (e.g., only cortisol), or academic performance without psychological outcomes were excluded.
Study design: Only empirical quantitative studies with original data were included, for example, randomized controlled trials (RCTs), non-randomized trials, cohort studies, and cross-sectional and longitudinal designs. Purely qualitative studies, case studies, and case series were excluded because of low generalizability. Previously published reviews, meta-analyses, and editorials were excluded, although their references were used to identify eligible primary studies. Only quantitative or mixed-methods studies reporting measurable psychological outcomes (e.g., perceived stress, anxiety, depression, and wellbeing) were included. Purely qualitative studies were excluded.
Publication type and language: Only peer-reviewed journal articles or academically recognized conference proceedings/repositories were accepted. Unpublished theses, internal reports, and non-peer-reviewed literature were excluded to ensure scientific quality. Articles in English or Spanish were accepted (studies in other languages were excluded because of a lack of translation). No minimum impact factor was required, but studies had to be accessible via the databases searched or Google Scholar (Table 1).

**Table 1:** Inclusion and exclusion criteria.

Inclusion criteria	Exclusion criteria
1. University students (under-/postgrad)	1. Preuniversity youth, non-students
2. Any study measuring or prescribing physical activity/exercise	2. Interventions without a PA component
3. Stress, anxiety, depression, and psychological wellbeing	3. Purely physical or academic outcomes
4. Quantitative originals (cross-sectional, cohort, quasi-exp., RCT)	4. Qualitative studies, reviews, and case reports
5. Peer-reviewed, 2020–2025, English or Spanish	5. Gray literature and other languages

### 2.4 Study selection process

The study selection process followed multiple phases consistent with the PRISMA flow. Initially, all references retrieved from the database searches were compiled, and duplicate entries were removed. Subsequently, titles and abstracts were screened to exclude clearly irrelevant records such as studies conducted in non-student populations or articles addressing physical activity without psychological measures. All records that appeared eligible or could not be confidently excluded were then examined in full during the eligibility phase. At this stage, the full text of each preselected study was independently reviewed by two reviewers, applying the inclusion and exclusion criteria described previously. For all studies excluded at this stage, the specific reasons were documented, including cases where the population fell outside the target range, stress was not measured, or the study design was inappropriate. Finally, the studies meeting all inclusion criteria were incorporated into the systematic review, and the progression of records through each phase was represented in a PRISMA flow diagram.

**Figure 1:**
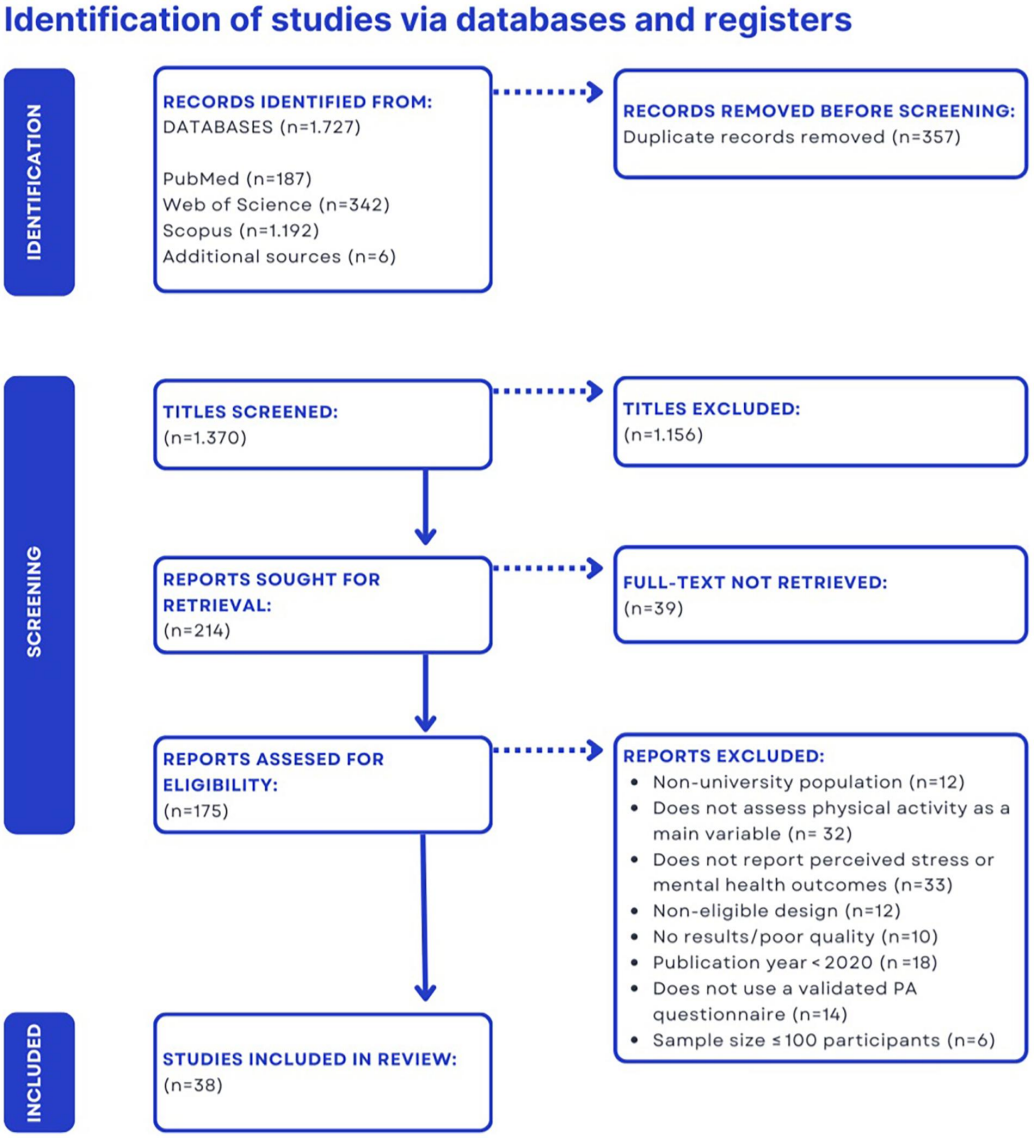
PRISMA flow diagram of the study identification, screening, eligibility, and inclusion process.

As illustrated in Figure 1, a total of 1,727 records (potential articles) were initially identified across databases and other sources. After removing 357 duplicates, 1,370 records were screened by title and abstract, of which 1,156 were excluded because of clear irrelevance. A total of 214 full-text articles were assessed for eligibility; however, 39 could not be retrieved in full text. Ultimately, 175 studies were evaluated in full, applying the preestablished inclusion criteria. Of these, 137 were excluded for various reasons (e.g., non-university population, lack of physical activity as a main variable, no reporting of stress or mental health outcomes, ineligible design, low quality, publication date prior to 2020 in some cases, use of non-validated physical activity instruments, or sample size ≤100; see Figure 1). As a result, 38 studies were included in the final qualitative synthesis (number indicated in the PRISMA diagram).

From each included study, key data were extracted using a standardized form, including (a) identification details (authors, year, and country), (b) sample characteristics (sample size, mean age, and percentage of women participants, etc.), (c) details of the physical activity measurement or intervention (type of activity, duration/frequency for interventions; measurement instruments in observational studies), (d) instruments used to assess perceived stress (e.g., Cohen's PSS) and mental health (e.g., anxiety/depression scales such as DASS-21, BDI, wellbeing, or academic stress scales), and (e) main results (association statistics such as correlation coefficients *r*, odds ratios, pre–post mean differences with confidence intervals, *p*-values, etc.), as well as any relevant qualitative findings reported.

Particular attention was paid to whether the studies reported mediation or moderation effects (e.g., variables that explain or influence the relationship between physical activity and mental health).

Given the expected diversity in outcome measures (perceived stress vs. various mental health scales) and study designs (cross-sectional vs. longitudinal vs. interventions), a narrative synthesis of findings was primarily used, complemented by summary tables. A qualitative and comparative description of the findings of each study was presented, organized thematically by outcome type or design similarity. Emphasis was placed on whether the studies found significant associations between physical activity and improved mental health outcomes, the magnitude and direction of these effects, and the consistency of evidence across studies. When multiple similar studies reported aligned findings, they were summarized together; when discrepancies or null results appeared, possible moderators were analyzed (e.g., methodological differences, study quality, and cultural context).

To facilitate comparative visualization, a synthesis table with the main characteristics and findings of each included study is provided. In the Results and Discussion sections, APA references are cited to support each statement with its original source.

## 3 Results

This section presents the empirical evidence obtained from the 38 studies included in the present review, whose main characteristics are summarized in Table 2. The table reports author and year, methodological design, sample characteristics, measurement instruments, and primary mental health outcomes for each investigation, thereby providing a concise mapping of the current literature. On the basis of this overview, the subsequent narrative is structured to distil the dominant trends—most notably the inverse association between physical activity and indicators of stress, anxiety, and depression—while relevant contextual moderators and methodological heterogeneity are critically appraised.

**Table 2:** Key characteristics of the studies included.

Author(s)	Title	Design	Sample	Instrument	Results
Lin et al. (13)	Association of 24-h Movement Guideline Adherence, Mental Health and Quality of Life in Young Adults: The Role of e-Health Literacy.	Cross-sectional study employing a mixed-effects statistical model	1,742 Chinese university students (mean age = 20.03 years; 68.6% women)	IPAQ-C, PSQI, SBQ, DASS-21, WHOQOL-Bref, eHLS-Web3.0	Greater adherence to a higher number of movement guidelines was associated with lower levels of depression, anxiety, and stress, as well as better quality of life. Meeting only the physical activity recommendation showed no significant association with mental health, whereas adherence to sleep and screen time guidelines did confer benefits. E-Health literacy acted as a mediator in the adherence–quality-of-life link and as a moderator in the adherence–mental health relationship.
Zhao et al. (14)	Exploring the Relationship between Physical Activity and Inhibitory Function in College Students with Depressive Symptoms through EEG	Cross-sectional study incorporating mediation analysis (EEG-based)	154 college students with depressive symptoms (mean age = 19.5 years; 59 men, 95 women)	BDI-II, PARS-3, Go/No-Go and Stroop tasks, resting-state EEG	Higher physical activity levels were associated with lower depression scores and superior response inhibition. EEG analyses identified delta and beta1 bands as partial mediators of this relationship. No moderating effect of gender was observed.
Zhang et al. (15)	Cross-sectional Association between 24-h Movement Guidelines and Depressive Symptoms in Chinese University Students	Cross-sectional study with multivariate analysis	1,793 Chinese university students from 10 universities (mean age = 20.7 ± 1.6 years; 63.6% women)	PHQ-9, IPAQ-SF, PSQI; sociodemographic covariates	Only 27.8% met all three 24-h movement recommendations (physical activity, sedentary time, and sleep). Students meeting none had 3.4 times higher odds of depressive symptoms (OR = 3.4, *p* < 0.001); meeting one guideline, OR = 2.7 (*p* < 0.001); meeting two, OR = 1.5 (*p* = 0.013). The association was stronger in men; for women, meeting two guidelines was not statistically significant. Overall, a greater adherence to the movement guidelines corresponded to a lower prevalence of depressive symptoms.
Ai et al. (16)	“Lifting More” Is Associated with Lower Risks of Depression in University Students	Cross-sectional study with multilevel multivariable regression	1,793 university students from 10 southern Chinese universities (mean age = 20.67 ± 1.61 years; 63.6% women) participating in the third wave of a pandemic-period cohort (August 2020)	Self-reported days/week of muscle-strengthening exercise (MSE); PHQ-9; IPAQ-SF; PSQI; demographic and behavioral covariates	Only 24.9% met WHO recommendations of ≥2 days/week MSE. Higher MSE frequency was significantly associated with lower depression scores (*β* = −0.17, 95% CI [−0.31, −0.03], *p* = 0.015). Non-adherence to MSE guidelines increased depressive symptom risk (*β* = 0.63, 95% CI [0.07, 1.19], *p* = 0.027) after controlling for sleep, overall physical activity, sedentary behavior, and sociodemographic factors.
Su et al. (17)	Physical Exercise, Sedentary Behaviour, Sleep and Depression Symptoms in Chinese Young Adults During the COVID-19 Pandemic: A Compositional Isotemporal Analysis	Cross-sectional study with compositional isotemporal substitution analysis (CoDA)	1,475 Chinese university students (mean age = 20.7 ± 1.6 years; 68% women)	IPAQ-SF; PSQI; PHQ-9; sociodemographic questionnaire	Replacing sedentary behavior with MVPA, sleep, or LPA for 5–15 min was linked to lower depressive symptoms; substituting sedentary time with MVPA produced the greatest improvement (−0.13 PHQ-9 points per 15 min). Shifting time from MVPA to sedentary behavior increased depressive symptoms, while replacing LPA with MVPA showed no significant effect. Increasing MVPA and reducing sedentary time thus appeared beneficial for mental health during the pandemic.
Silva et al. (18)	Mental Health in Young Adult University Students During COVID-19 Lockdown: Associations with Physical Activity, Sedentary Behaviors and Sleep Quality	Cross-sectional observational study	549 Portuguese university students (18–30 years; 57.7% men; 73% sport-science majors; 91% physically active during lockdown)	DASS-21; IPAQ-SF; self-reported sitting time, screen use, social media use; PSQI	Poor sleep quality, prolonged sitting, and computer use for study/work were linked to higher depression, anxiety, and stress. Vigorous physical activity showed an inverse (protective) association. Women exhibited poorer mental health and sleep outcomes than men. Findings support promoting vigorous exercise, reducing sedentary behavior, and improving sleep quality to safeguard student mental health during lockdowns.
Litwic-Kaminska and Kotysko (19)	Sleep Quality of Student Athletes and Non-Athletes: The Role of Chronotype, Stress and Life Satisfaction	Cross-sectional observational study with mediation analysis	335 Polish university students—207 athletes (mean age = 21.14 ± 1.77 years; 73% men) and 128 low-active non-athletes (mean age = 21.52 ± 2.94 years; 23.4% men)	MEQ (chronotype); PSS-10 (perceived stress); SWLS (life satisfaction); PSQI (sleep quality); IPAQ-SF (physical activity screening)	Athletes showed better sleep (lower PSQI), lower stress, and higher life satisfaction than non-athletes (*p* < 0.001), along with greater morningness preference. Chronotype affected sleep quality both directly and indirectly via perceived stress (partial mediation), a pattern observed in athletes and non-athletes alike. Morning preference and reduced stress thus emerged as key contributors to superior sleep quality, with regular athletic activity likely acting as a stress buffer.
Zhang et al. (20)	The Relationship between Physical Activity Intensity and Subjective Well-Being in College Students	Cross-sectional observational study	723 university students at a public university in Beijing (mean age = 19.3 years; 59% men)	Chinese IPAQ-SF; SWLS; SHS; SPANE; covariates: age, sex	Vigorous physical activity was the only significant predictor of life satisfaction (*β* = 0.13, *p* < 0.001). Both vigorous- and moderate-intensity activity correlated positively with happiness and positive affect and negatively with negative affect, whereas walking showed no significant links to any wellbeing component. Effects were stronger for affective (emotional) than cognitive (life satisfaction) domains, underscoring the role of moderate-to-vigorous activity in enhancing students’ positive emotions.
Ugwueze et al. (21)	Relationship between Physical Activity Levels and Psychological Well-Being Among Male University Students in South-East Nigeria: A Cross-Sectional Study	Cross-sectional observational study	243 male university students (18–30 years; mean age = 24.9 ± 7.61 years) at a public university in south-eastern Nigeria	IPAQ-SF (physical activity); Ryff’s Psychological Wellbeing Scale (42-item, six-dimension PWB); anthropometrics and sociodemographic questionnaire	PA distribution: 16.0% low, 64.2% moderate, 19.8% high (≥3,000 MET-min/week). Higher total PA correlated with greater overall psychological wellbeing (*β* = 0.13, *p* < 0.05) and with auto-acceptance (*β* = 0.13), positive relations (*β* = 0.16), and purpose in life (*β* = 0.39). Vigorous PA was negatively associated with personal growth (*β* = −0.28). PA accounted for 2.5% of the variance in positive relations, 1.2% in purpose in life, and 2.0% (negative) in personal growth, indicating modest but significant links between physical activity and multiple facets of psychological wellbeing.
Moriarty et al. (22)	The Relationship between Psychological Stress and Healthy Lifestyle Behaviors during COVID-19 among Students in a US Midwest University	Cross-sectional online survey	550 university students (mean age = 21.3 ± 3.8 years; 74.2% women; 94.4% white; Midwestern USA)	PSS-4 (perceived stress); IPAQ-SF (physical activity); adapted NIH COVID-19 Lifestyle Change items (exercise, sleep, and social connection)	60.9% screened high for stress (PSS-4 ≥8). During the pandemic, 39.1% exercised less and 21.2% slept less. Lower exercise and sleep time were each associated with higher perceived stress (*p* < 0.001). Reductions in exercise (*β* = 0.85) and sleep (*β* = 1.86) remained significant predictors after adjusting for gender, employment, and income; being female, unemployed, or low income also elevated stress. The model explained 19.6% of stress variance, suggesting that maintaining adequate exercise and sleep may buffer psychological stress in college students during crises.
Li et al. (23)	The Mediating Role of Resilience in the Effects of Physical Exercise on College Students’ Negative Emotions during the COVID-19 Epidemic	Cross-sectional SEM mediation study	1,214 home-confined college students (mean age = 19.99 ± 1.73 years; 41.7% men) from three universities in Shandong, Liaoning, and Jilin (China)	Chinese 8-item Physical Exercise Questionnaire; 21-item Depression–Anxiety Scale; Chinese CD-RISC (resilience)	More exercise correlated with fewer negative emotions (*r* = −0.25) and higher resilience (*r* = 0.47); resilience also inversely related to negative emotions (*r* = −0.33). SEM showed exercise directly reduced negative emotions (*β* = −0.14) and indirectly did so via increased resilience (exercise → resilience *β* = 0.51; resilience → negative emotions *β* = −0.29); resilience mediated ∼50% of the total effect. Thus, physical activity lessens distress both directly and by boosting resilience during COVID-19 confinement.
Elliott et al. (24)	Participation in Higher Intensity Physical Activity Predicts Lower Depressive Symptom Incidence in College Students	Cross-sectional online survey	1,738 college students (mean age = 20.5 ± 1.6 years; 63.5% women; 72.8% white; Northeastern USA)	GPAQ (moderate and vigorous PA); single-item strength training days; CESD-7 (depression); self-reported restful sleep days and GPA	Vigorous PA minutes (*β* = −0.11, *p* < 0.001) and strength training frequency (*β* = −0.11, *p* < 0.001) predicted fewer depressive symptoms, whereas moderate PA was non-significant. Insufficient sleep, lower GPA, and non-heterosexual orientation were linked to higher depression. The VPA + ST model accounted for 15.7% of variance, highlighting higher-intensity exercise as a protective factor for college students’ mental health.
Baranauskas et al. (25)	Mental Health and Physical Activity of Female Higher Education Students during the COVID-19 Pandemic: A Comparative Cross-Sectional Study from Lithuania	Comparative cross-sectional survey	1,231 women students aged 18–29 years (630 medical/health sciences; 601 other disciplines) in Lithuania	HADS (anxiety, depression); PHQ-15 (somatic symptoms); Baecke PA Questionnaire (work, sport, leisure)	Severe anxiety: 51.9% (higher in non-biomedical students 59.6% vs. 44.6%); severe depression: 11% (14.1% vs. 7.9%); severe somatization: 21.3% (higher in non-biomedical). Biomedical students reported more PA across all domains. Greater sport-related PA was associated with lower depression severity in both groups (adjusted OR ≈ 0.4); habitual PA showed no significant link with anxiety. The results suggest sport PA may help reduce depressive symptoms, while non-biomedical students appear more vulnerable to mental health issues.
Reyes-Molina et al. (26)	Association between the Physical Activity Behavioral Profile and Sedentary Time with Subjective Well-Being and Mental Health in Chilean University Students during the COVID-19 Pandemic	Cross-sectional correlational study	382 university students (mean age = 22.4 ± 0.19 years; 66% women; Chile)	IPAQ-SF (physical activity and sedentary time); Pemberton Happiness Index; GHQ-12; 4-item PHQ-9 (sleep, fatigue, appetite, and concentration)	Inactive + sedentary students showed the poorest subjective wellbeing, positive affect, and general mental health. Being physically active and non-sedentary predicted better mental health than being active but sedentary, highlighting sedentary lifestyle as a key negative factor for Chilean students’ mental health during COVID-19.
Porter et al. (27)	24-H Movement Guideline Adherence and Mental Health: A Cross-Sectional Study of Emerging Adults with Chronic Health Conditions and Disabilities	Cross-sectional study	17,874 emerging adults (mean age = 21.6 years; 65.2% women) from 20 Canadian post-secondary institutions; 3,336 reported chronic conditions/disabilities (CCD)	IPAQ (physical activity); modified ISAT (screen and sitting time); sleep timing questionnaire; K10 (psychological distress); WEMWBS (mental wellbeing)	Students with CCD were less likely to meet the 24-h movement guidelines. Adhering to the PA, sleep, and sedentary behavior recommendations was associated with lower distress and higher wellbeing in both CCD and non-CCD groups, with the greatest mental health benefit when all three guidelines were met. Those with multimorbidities or developmental disabilities showed the lowest adherence rates.
Valentim et al. (28)	The Relation between Lifestyles and Positive Mental Health in Portuguese Higher Education Students	Cross-sectional, descriptive, correlational, multicenter survey	3,647 higher-education students from 20 Portuguese institutions (mean age = 23 years; 78.8% women)	Self-administered questionnaire (sociodemographics, clinical data, and lifestyle); Portuguese Positive Mental Health Questionnaire (PMHQ)	Four clusters of positive mental health emerged. Students in the highest-PMH cluster exhibited healthier lifestyles: better sleep quality, more physical activity, healthier diet, lower medication/illicit substance use, and greater satisfaction with close relationships. Findings underscore a positive association between healthy lifestyle habits and higher positive mental health among Portuguese university students.
Rodríguez-Romo et al. (29)	Physical Activity and Mental Health in Undergraduate Students	Cross-sectional descriptive study	847 university students (mean age ≈ 21.6 years) from public and private universities in Madrid, Spain	GPAQ-v2 (total, leisure-time, occupational PA); GHQ-12 (mental health)	Greater total PA was associated with better mental health. High leisure-time PA and moderate occupational PA reduced vulnerability to mental health problems by 47% and 51%, respectively, underscoring the protective value of both vigorous leisure activity and moderate work-related activity for students’ wellbeing.
Çerezci-Duygu et al. (30)	Relationship between Physical Activity Level and Stress Perception: Exploring Factors During COVID-19 Pandemic	Cross-sectional study	444 university students (mean age = 21 ± 2.95 years; 81.3% women; Turkey)	IPAQ-SF (physical activity); Perceived Stress Scale; PA barriers and motivators questionnaires	Total, vigorous, and walking PA levels were each inversely related to perceived stress. Financial insufficiency became a less-reported barrier during the pandemic, while advice from health professionals grew as a motivator. The results indicate that boosting physical activity may help reduce stress among university students amid COVID-19 restrictions.
Johannes et al. (31)	Relationship between Psychosocial Factors and Physical Activity among Undergraduate Students from a South African University	Cross-sectional quantitative study	534 undergraduates (mean age ≈ 21.1 years; 53.6% women) at a historically disadvantaged South-African university	IPAQ-SF (physical activity); DASS-21 (depression, anxiety, and stress); PALMS (motivation); PSS-Family and PSS-Friends (social support)	29% inactive, 31.1% minimally active, 39.9% sufficiently active. Physical activity showed small positive correlations with stress (*r* = 0.11) and anxiety (*r* = 0.10). Motivation factors—psychological condition and others’ expectations—were significant correlations, whereas perceived social support from family or friends was not.
Deng et al. (32)	The Association between Physical Activity and Anxiety in College Students: Parallel Mediation of Life Satisfaction and Self-Efficacy	Cross-sectional study	358 Chinese college students (mean age = 20.88 years; 52% men)	PARS-3 (physical activity); CSLSS (life satisfaction); GSES (self-efficacy); GAD-7 (anxiety)	Greater physical activity was linked to lower anxiety. Life satisfaction and self-efficacy each partially mediated this relationship, jointly explaining ≈ 55% of the total effect. Thus, physical activity appears to lessen anxiety by simultaneously enhancing both life satisfaction and self-efficacy, with no moderating role of gender.
Ofili et al. (33)	Physical Activity and Depressive Symptoms during the Fifth Wave of the COVID-19 Pandemic: Implications for Public Policy and Administrators	Cross-sectional observational study	383 university students (mean age = 23.08 years; 57.2% women) at the University of Nigeria	IPAQ-SF (physical activity); PHQ-9 (depressive symptoms)	31.3% had low, 63.2% moderate, and 5.5% high PA. Overall, 77% screened positive for depression (29% mild, 12% severe). Physical activity showed a strong inverse correlation with depressive symptoms (*r* = −0.90). PA levels did not vary by gender, residence, age, or year of study, but depressive symptoms were higher in women. Promoting PA may help reduce depression among Nigerian students during pandemic waves.
Liu et al. (34)	The Influence of Exercise Adherence on Peace of Mind among Chinese College Students: A Moderated Chain Mediation Model	Cross-sectional moderated chain mediation study	1,520 college students (mean age = 20.56 years; 41.7% men) from four western-China universities	Exercise Adherence Questionnaire; Meaning in Life Questionnaire; Brief Self-Control Scale; Peace of Mind Scale; Self-Acceptance Questionnaire	Exercise adherence directly increased peace of mind (*b* = 0.087). Meaning in life and self-control each independently—and sequentially—mediated this relationship, with self-acceptance, amplifying the link between meaning in life and self-control at higher acceptance levels. Promoting exercise adherence alongside life meaning, self-control, and self-acceptance may enhance students’ peace of mind.
Hong et al. (35)	The Association between Physical Fitness and Mental Health among College Students: A Cross-Sectional Study	Cross-sectional study	6,724 college students (aged 16–24 years) at the Changzhou Vocational Institute of Engineering, China	Chinese University Students Physique Test (physical fitness); University Personality Inventory (UPI)—symptoms subscales (somatic, schizophrenia, depression, and neuroticism/persecutory beliefs)	Higher fitness levels (“pass” or “good”) were linked to lower scores on somatic symptoms, schizophrenia, depression, and neuroticism/persecutory beliefs. Associations remained after adjusting for age, sex, BMI, survey year, faculty, and vision, highlighting poorer mental health profiles among students with low physical fitness.
Mu et al. (36)	Influence of Physical Exercise on Negative Emotions in College Students: Chain Mediating Role of Sleep Quality and Self-Rated Health	Cross-sectional study	30,475 college students in China (aged 16–24 years)	PARS-3 (physical activity); DASS-21 (stress, anxiety, and depression); PSQI (sleep quality); SF-12 self-rated health	Some 77.6% engaged only in low-intensity exercise. Higher physical activity scores correlated with lower stress, anxiety, and depression and with better self-rated health. Sleep quality and self-perceived health each partly mediated the exercise–emotion link, suggesting that improving sleep and health perception can amplify the emotional benefits of physical activity for students.
Wang et al. (37)	How to Reduce Anxiety Symptoms through Individual Sport in Youth: An 8-Month Longitudinal Study	8-Month longitudinal study	163 university students (81.6% men; Guilin University of Electronic Technology, China)	IPAQ (physical activity, sedentary time, and sleep); table tennis skill test (Nine-Level System); GSES; RSES; CD-RISC-25; HAM-A	Self-efficacy and self-esteem rose over the 8 month-period, whereas resilience and overall anxiety did not change significantly. Higher table tennis skill buffered the development of anxiety symptoms in students whose self-esteem declined. Changes in self-efficacy, self-esteem, and sedentary hours predicted changes in anxiety, highlighting individual sports (e.g., table tennis) as a potential mental health protective factor for youth.
Jelleli et al. (38)	Examining the Interplay between Physical Activity, Problematic Internet Use and the Negative Emotional State of Depression, Anxiety and Stress: Insights from a Moderated Mediation Path Model in University Students	Cross-sectional moderated-mediation study	976 Tunisian university students (mean age = 20.8 ± 1.6 years; 52.5% women)	CIUS (problematic internet use); IPAQ (physical activity); DASS-21 (depression, anxiety, and stress)	Physical activity was inversely related to problematic internet use (*r* = −0.49) and to stress, anxiety, and depression (*r* = −0.52), while PIU showed a positive link with emotional symptoms (*r* = 0.54). PA weakened the effect of PIU on negative emotions, and this moderation was not influenced by gender. Promoting regular physical activity may therefore buffer the mental health impact of problematic internet use among university students.
Wu and Zhou (39)	Effectiveness of Acute Aerobic Exercise in Regulating Emotions in Individuals with Test Anxiety	Randomized controlled experiment	44 university students with high test anxiety (22 exercise and 22 control)	30 min moderate-intensity aerobic session vs. quiet control; TAS and TAI (test anxiety), PANAS and POMS (affect), and EEG Frontal Alpha Asymmetry (FAA)	Single aerobic bout significantly reduced test anxiety scores and negative moods (tension, depression, anger, and confusion) and increased left-frontal FAA, a marker of better emotion regulation. Reductions in the negative affect correlated with FAA shifts, indicating that acute aerobic exercise can quickly alleviate exam-related anxiety through neuro-affective mechanisms.
Zhang et al. (40)	Physical Activity, Weight Management, and Mental Health during COVID-19 Lockdown: A Cross-Sectional Study of Healthcare Students in China	Cross-sectional survey	1,216 health-science students (Gansu Province, China)	IPAQ-SF (physical activity); 11-item weight control behavior questionnaire; WHO-5 (wellbeing)	45% reported low PA and 54.6% scored low on wellbeing. Absence of prepandemic exercise habits and poorer weight management behaviors predicted low PA, while greater PA related to healthier weight status and higher wellbeing. The findings highlight the need to promote physical activity among healthcare students to support both psychological wellbeing and weight control practices.
Barbosa et al. (41)	Sedentary Behavior Is Associated with the Mental Health of University Students during the COVID-19 Pandemic, and Not Practicing Physical Activity Accentuates Its Adverse Effects: A Cross-Sectional Study	Multicenter cross-sectional survey	8,650 university students from eight public universities in Brazil	DASS-21 (anxiety and depression); adapted IPAQ items (sedentary behavior); MVPA-per-sedentary-hour ratio	≥9 h/day sedentary time raised odds of anxiety (OR = 1.37) and depression (OR = 1.61). Doing < 2.5 min moderate-to-vigorous PA per sedentary hour also increased anxiety (OR = 1.44) and depression (OR = 1.74). Sufficient PA attenuated the mental health risks linked to high sedentary behavior, underscoring the need for university policies that curb sitting time and promote activity.
Zhu et al. (42)	The Relationship between Exercise Habits and Mental Health among University Students in China: A Cross-Sectional Study Based on Instrumental Variable Analysis	Cross-sectional IV analysis	1,120 university students from three Chinese universities (mean age = 21.6 years; 58.5% women)	Self-Report Habit Index (exercise habits); PANAS (positive affect); Satisfaction with Life Scale; Short Index of Self-Actualization; IV = dorm-to-sports-field distance	Stronger exercise habits predicted a higher positive affect (*β* = 0.263), life satisfaction (*β* = 0.151), and self-actualization (*β* = 0.102) in the IV model, with larger effects than OLS, implying a causal benefit of habitual exercise for mental health. Universities should foster consistent exercise routines to enhance students’ wellbeing.
Ye et al. (43)	The Effect of Physical Activity on Depression: A Lagged Mediation Study of School Burnout	Two-wave longitudinal study (one semester)	305 male undergraduates (18–21 years) at Wuhan University of Science & Technology, China	Godin–Shephard Leisure-Time PA Questionnaire; MBI-SS (school burnout); PHQ-9 (depression)	Greater physical activity predicted lower burnout and depression at both time points. School burnout partially mediated the PA–depression link, with end-semester burnout as the stronger mediator. High-intensity PA conferred the largest protective effect, suggesting that regular vigorous activity can help prevent school burnout and depressive symptoms in students.
Wang et al. (44)	Correlation Between Physical Exercise Levels, Depressive Symptoms, and Sleep Quality in College Students: Evidence from Electroencephalography	Cross-sectional study	342 college students (17–25 years; Songjiang University Town, Shanghai, China)	PARS-3 (physical activity); BDI-II (depression); PSQI (sleep quality); 16-channel resting-state EEG	Poorer sleep quality correlated with more depressive symptoms (*r* = 0.55), while higher physical activity scores related to better sleep (*r* = −0.16) and less daytime dysfunction. Sleep quality mediated ≈41% of the PA–depression relationship. Greater exercise was also linked to lower theta-band power at F8, consistent with improved mood and sleep.
Wang et al. (45)	Association between Social Media Use, Physical Activity Level, and Depression and Anxiety among College Students: A Cross-Cultural Comparative Study	Cross-sectional comparative survey with mediation/moderation models	1,500 undergraduates (aged 18–25 years; 52% women) from 10 Chinese universities	SMUIS (social media use); IPAQ (physical activity); DASS-21 (depression, anxiety); PSQI (sleep quality); Cultural Values Scale (collectivism)	Heavier social media use correlated with more depression (*r* = 0.28) and anxiety (*r* = 0.31), with effects stronger in women. Greater physical activity predicted lower depression (*β* = −0.18) and anxiety (*β* = −0.15) and blunted the mental health impact of social media use. Poor sleep partly mediated the social media–depression link (∼30%), while collectivist values buffered negative effects. Marked gender and regional differences highlight the need for tailored, context-specific interventions.
Yu et al. (46)	The Pathway Relationship Between Physical Activity Levels and Depressive Symptoms in University Students Mediated by Cognitive Flexibility	Cross-sectional study	2,537 university students (17–26 years; Shanghai, China)	PARS-3 (physical activity); PHQ-9 (depressive symptoms); Cognitive Flexibility Inventory—controllability and alternatives	Exercise intensity correlated negatively with depressive symptoms (*r* = −0.10, *p* < 0.01) and positively with controllability (*r* = 0.11, *p* < 0.01). Controllability partly mediated the PA–depression link (total effect = −0.354; direct = −0.220; indirect = −0.134), explaining ∼38% of the association. Higher-intensity activity appears to reduce depression partly by strengthening cognitive flexibility (controllability).
Cheng et al. (47)	Relationships between Exercise Components and Social Anxiety Levels Among Chinese College Students	Cross-sectional study	844 college students (≈54.7% men) from six Qingdao universities (China)	PARS-3 (exercise frequency, intensity, and duration); LSAS (social anxiety); demographic survey	Moderate-to-high-intensity PA is linked to lower social anxiety. Exercise duration ≥1 h and frequency ≥1–2 sessions/week were the strongest protective factors, whereas intensity contributed less. Benefits plateaued beyond a certain exercise threshold, indicating that programs should prioritize sustained and regular activity to reduce social anxiety.
Çakir et al. (48)	An Evaluation of Physical Activity Levels and Mental Health Among Young People: A Cross-Sectional Study	Cross-sectional survey	427 university students (Recep Tayyip Erdoğan University, Turkey)	IPAQ-SF (physical activity); WEMWBS-SF (mental wellbeing); Short Psychological Resilience Scale; Psychological Vulnerability Scale	32.7% of students were inactive, 46.3% were minimally active, and 20.8% were health-enhancing physically active (HEPA). HEPA students showed higher psychological resilience, better wellbeing, and lower vulnerability than inactive/minimally active peers. Walking was the strongest predictor of wellbeing and resilience—especially when combined with moderate activity—whereas vigorous exercise had no significant mental health effect. Women engaged less in vigorous activity and reported greater psychological vulnerability.
Li et al. (49)	Physical Activity and Happiness of College Students: Chain Mediating Role of Exercise Attitude and Sleep Quality	Cross-sectional chain-mediation study	1,308 university students (18–24 years) from four provinces in China (Sichuan, Chongqing, Henan, and Heilongjiang)	IPAQ-SF (physical activity); Exercise Attitude Scale; Pittsburgh Sleep Quality Index; Overall Happiness Scale	Greater physical activity predicted higher happiness. Exercise attitude was a significant mediator, and a sequential path—PA → exercise attitude → sleep quality → happiness—also emerged; sleep quality alone did not mediate independently. Promoting positive attitudes toward exercise and better sleep may therefore enhance the happiness benefits of physical activity in college students.
Li et al. (50)	Cross-Sectional Associations of Physical Activity and Sleep with Mental Health among Chinese University Students	Large cross-sectional survey	30,475 students from 104 universities across 31 Chinese provinces	IPAQ-SF (physical activity); PSQI (sleep duration and quality); CES-D (depressive symptoms); GAD-7 (anxiety); health behavior and sociodemographic questionnaire	77.6% engaged only in light PA; 39.5% reported insufficient sleep and 16.7% poor sleep quality. High-risk depression (CES-D) affected 10% and high-risk anxiety (GAD-7) 23.3%. Moderate-to-vigorous PA and sufficient, good-quality sleep were each linked to lower depression risk; adequate sleep also related to lower anxiety. An interaction indicated that the mental health benefit of PA was stronger when sleep was sufficient and of high quality. The findings support combined PA-and-sleep interventions to improve students’ mental health.

IPAQ-C, International Physical Activity Questionnaire (Chinese); IPAQ-SF, International Physical Activity Questionnaire (Short Form); IPAQ v2, International Physical Activity Questionnaire (version 2); GPAQ, Global Physical Activity Questionnaire; PARS-3, Physical Activity Rating Scale-3; SBQ, Sedentary Behavior Questionnaire; ISAT (mod.), International Sedentary Assessment Tool (modified); MVPA, Moderate-to-Vigorous Physical Activity; LPA, Light Physical Activity; PSQI, Pittsburgh Sleep Quality Index; WHOQOL-BREF, World Health Organization Quality of Life (brief version); SF-12, 12-Item Short Form Health Survey; WHO-5, WHO–Five Well-Being Index; DASS-21, Depression, Anxiety and Stress Scales (21 items); PHQ-9, Patient Health Questionnaire-9; PHQ-15, Patient Health Questionnaire-15; HADS, Hospital Anxiety and Depression Scale; GHQ-12, General Health Questionnaire-12; K10, Kessler Psychological Distress Scale (10 items); GAD-7, Generalized Anxiety Disorder-7; WEMWBS, Warwick–Edinburgh Mental Well-Being Scale; WEMWBS-SF, Warwick–Edinburgh Mental Well-Being Scale (short form); PMHQ, Positive Mental Health Questionnaire; Ryff’s PWB, Psychological Well-Being Scale; SWLS, Satisfaction With Life Scale; SHS, Subjective Happiness Scale; SPANE, Scale of Positive and Negative Experience; POMS, Profile of Mood States; PANAS, Positive and Negative Affect Schedule; LSAS, Liebowitz Social Anxiety Scale; HAM-A, Hamilton Anxiety Rating Scale; PSS-10, Perceived Stress Scale (10 items); PSS-4, Perceived Stress Scale (4 items); PSS Family, Perceived Social Support (Family); PSS Friends, Perceived Social Support (Friends); MEQ, Morningness–Eveningness Questionnaire; GSES, General Self-Efficacy Scale; RSES, Rosenberg Self-Esteem Scale; CD-RISC-25, Connor–Davidson Resilience Scale (25 items); CFI, Cognitive Flexibility Inventory; UPI, University Personality Inventory; TAS, Test Anxiety Scale; TAI, Test Anxiety Inventory; EEG, Electroencephalography; FAA, Frontal Alpha Asymmetry; CIUS, Compulsive Internet Use Scale; SMUIS, Social Media Use Integration Scale; MBI-SS, Maslach Burnout Inventory–Student Survey; SISA, Short Index of Self-Actualization; IV, Instrumental Variables; OLS, Ordinary Least Squares; eHLS-Web3.0, eHealth Literacy Scale (Web 3.0).

### 3.1 Characteristics of the included studies

The 38 studies included in this review encompass a wide range of designs, populations, and outcomes (Table 2). More than 80% of them (approximately 31 studies) employed a cross-sectional design, relying on surveys or one-time measurements to examine associations between levels of physical activity and psychological variables. A smaller portion consisted of longitudinal studies, which tracked participants over several weeks or months, and a few included experimental or intervention-based approaches. For instance, an acute experiment assessed the immediate emotional effects of a short aerobic session on students experiencing exam anxiety, finding improved emotional regulation following the exercise (39). Only one true randomized controlled trial was identified, and it was technically a one-session experiment; the other interventions were quasi-experimental or pre–post studies without formal control groups.

Collectively, the included studies evaluated over 20,000 university students, with individual sample sizes ranging from 154 to more than 1,700. The studies were conducted in diverse geographic locations, with China contributing the largest number, followed by the United States and Canada, several European countries (such as Spain, Portugal, and Lithuania), African nations (including Nigeria, South Africa, and Tunisia), and a few from the Middle East. While some studies focused on specific populations—such as health science students, women students, or students with chronic illnesses or disabilities—most investigated general university populations. The average age of participants typically ranged between 19 and 22 years, and the percentage of women participants varied widely, from approximately 50% to 100%, depending on the sample and research focus.

### 3.2 Measurement instruments and key variables

To assess physical activity, most studies used validated self-report instruments such as the International Physical Activity Questionnaire (IPAQ, in its various versions), the Physical Activity Rating Scale (PARS-3, especially in Chinese studies), or self-reported compliance with the 24-h Movement Guidelines, which collectively consider physical activity, sedentary behavior, and sleep. For perceived stress, the most frequently used instrument was the PSS, either in its 10- or 14-item version, along with some academic stress-specific adaptations. Symptoms of anxiety and depression were commonly measured with standardized tools such as DASS-21, the Beck Depression Inventory-II (BDI-II), and the Patient Health Questionnaire-9 (PHQ-9). Positive mental health constructs like life satisfaction or psychological wellbeing were also assessed using established scales. One study from China incorporated the culturally adapted concept of “peace of mind.” In addition, several studies included mediating or moderating variables such as sleep quality (measured using the Pittsburgh Sleep Quality Index), psychological resilience (with tools like the Connor–Davidson Resilience Scale), academic burnout (MBI-SS), or problematic internet use to better understand the pathways through which physical activity may influence mental health outcomes.

### 3.3 Main associations observed

The evidence across cross-sectional studies consistently indicated that university students who were more physically active tended to report better mental health indicators. Specifically, these students exhibited lower levels of perceived stress, anxiety, and depressive symptoms and higher levels of psychological wellbeing compared with their less active peers. For example, some studies (13, 15, 21, 29) confirmed these associations. A Chinese study involving more than 1,700 participants found that adherence to all three recommendations in the 24-h Movement Guidelines—adequate physical activity, sufficient sleep, and reduced sedentary time—was significantly associated with fewer symptoms of depression, anxiety, and stress, as measured by DASS-21. Regular participation in physical activity is linked to lower academic stress and higher life satisfaction (18, 28). In Portuguese samples, healthier lifestyle habits—including exercise—correlated positively with subjective wellbeing (28).

### 3.4 Specific findings on perceived stress, anxiety, and depression

Perceived stress was a central outcome across the evidence base. Earlier work already showed that physical activity and reduced sedentary behavior were independently linked to lower stress levels (5). The present review incorporates newer studies that confirm and extend this observation. Among Turkish undergraduates during the COVID-19 pandemic, higher PARS-3 scores were inversely related to perceived stress on the PSS, and physical activity still explained approximately 20% of the variance after controlling for gender, BMI, and internet use (30). Similar results emerged in a US cohort, in which regular vigorous exercise predicted both fewer depressive symptoms and lower perceived stress, underscoring the importance of activity frequency and intensity for mood regulation (24).

Comparable protective patterns were observed for depression and anxiety. Data from China (15), Nigeria (21), Spain (29), and Lithuania (19) consistently indicated that physically active students scored lower on depression and anxiety scales than their sedentary peers, with effects persisting after adjustment for confounders. In the Spanish sample, for instance, physical activity independently predicted better general mental health, and active participants had roughly 30%–40% lower odds of anxiety or depression (29). A clear dose–response trend also emerged: failing to meet any component of the 24-h movement guidelines was associated with a 3.4-fold higher risk of depression compared with adhering to all three components (15).

### 3.5 Mediators and moderators

Several studies examined the mechanisms through which physical activity may impact mental health. Sleep quality emerged as a particularly important mediator. For instance, Mu et al. found that the beneficial effects of physical activity on reducing negative emotions were partly explained by improvements in sleep and higher self-rated health. Wang et al. similarly found that sleep quality mediated approximately 40% of the relationship between physical activity and depression and also noted EEG changes that corresponded with improved mood. Psychological resilience also played a mediating role. Li et al. documented that resilience accounted for nearly 50% of the protective effect of physical activity during the pandemic, as more resilient students reported fewer negative emotional outcomes. In Tunisia, Jelleli et al. developed a moderated mediation model showing that exercise mitigated the negative effects of problematic internet use on mental health. Gender was also explored as a moderator, with stronger associations between exercise and reduced depression reported among men (15). However, no such effect was observed in a study on cognitive control (8).

### 3.6 Longitudinal and experimental evidence

Beyond cross-sectional evidence, several longitudinal and experimental studies strengthen the case for a causal link between physical activity and mental health outcomes in university students. An 8-month follow-up of 160 students showed that regular participation in an individual sport produced sustained reductions in anxiety relative to a non-exercising control group (37). A two-wave delayed-mediation design found that baseline physical activity predicted lower academic burnout, which, in turn, reduced depressive symptoms by the end of the semester, with burnout acting as the mediator (43). An acute exercise experiment demonstrated that a single bout of moderate aerobic activity immediately lowered tension and anxiety in exam-anxious students (39). Complementing these results, data from students with chronic conditions or disabilities indicated that meeting all components of the 24-h Movement Guidelines was associated with substantially better mental health outcomes, highlighting the value of adapted activity for medically vulnerable populations (27).

A few investigations reported null or more nuanced results that warrant attention. One study (13) showed that meeting only the physical activity recommendation—without also achieving guideline targets for sleep and screen time—did not yield significant improvements in mental health indicators. The greatest benefits appeared when adequate sleep accompanied regular activity. In the same vein, the relationship between exercise and perceived stress was fully mediated by sleep quality in a Portuguese sample (18), indicating that physical activity did not directly predict stress reduction once sleep was taken into account. Additional nuance comes from evidence that, among students with depression, physical activity improved inhibitory control and altered EEG markers of frontal activity (14), suggesting effects that extend beyond self-report to neurophysiological domains of mental health.

### 3.8 Summary

In summary, the findings from this systematic review consistently support the conclusion that higher levels of physical activity are associated with better mental health in university students. Although the magnitude of the associations is typically small to moderate (e.g., correlations around *r* = 0.2–0.4; odds ratios between 2 and 3 for mental disorder risk in inactive vs. active students), the consistency across studies and contexts is compelling. Mediation analyses highlight the importance of sleep quality, resilience, and academic burnout as key pathways through which physical activity exerts its benefits. In addition, the inclusion of several longitudinal and experimental studies strengthens the argument for a possible causal relationship. Notably, no study reported any detrimental effects of physical activity on mental health. Rather, being active—even in challenging contexts such as the COVID-19 pandemic—appeared consistently linked to more favorable psychological outcomes. These findings reinforce the importance of promoting physical activity as a potentially effective strategy for stress reduction and mental health promotion in university populations.

## 4 Discussion

The findings of this systematic review provide robust evidence that physical activity is consistently associated with lower perceived stress and better mental health outcomes in university students. Across the included studies, the magnitude of associations typically ranged from small to moderate (correlations ≈0.10–0.40; odds ratios between 1.5 and 3.4). Nevertheless, the consistency of these effects across diverse regions, methodologies, and sample characteristics reinforces their practical relevance. Importantly, the strength of the associations varied depending on study design: Longitudinal and intervention studies generally reported more stable or larger effects than cross-sectional designs, suggesting that temporal increases in physical activity may precede improvements in stress and emotional wellbeing.

### 4.1 Sedentary behavior as a risk factor

It is also important to note the conceptual distinction between physical activity and sedentary behavior, as individuals may meet activity recommendations while still accumulating prolonged sitting time, which carries independent mental health risks (3, 13). Physiologically, excessive sedentarism has been linked to impairments in glucose metabolism, reduced neuroplasticity markers such as brain-derived neurotrophic factor (BDNF), and dysregulation of the stress–response system. Psychosocial pathways include lower social interaction and cognitive disengagement. In interpreting the psychological mechanisms identified in this review, it should be acknowledged that some mediators such as resilience or self-efficacy are supported by a limited number of studies and mostly correlational designs. Therefore, these explanations should be considered preliminary in nature. Integrating these mechanisms with established biological pathways provides a more comprehensive account of how physical activity and sedentarism jointly influence mental health in university students (21, 29, 33).

A key contribution of the present synthesis is the clarification of population-specific variability. Associations tended to be stronger in students with preexisting psychological symptoms—such as those reporting depressive symptomatology or high academic stress—compared with general non-clinical student samples (30). Subgroup analyses also revealed that activity type mattered: Vigorous activity and multimodal movement patterns (including meeting sleep and sedentary behavior recommendations) frequently showed stronger protective effects than light activity or physical activity considered in isolation (17). These nuances underscore that mental health benefits may not be uniform across all student groups and that both the intensity and the behavioral context of activity influence outcomes.

A particularly salient finding is the consistent link between physical inactivity, prolonged sitting, and poorer mental health outcomes. Across samples from several countries, students classified as inactive were 1.5–3 times more likely to exhibit elevated stress, anxiety, or depressive symptoms than their more active peers (13, 15, 21). This pattern indicates that, much like its impact on physical health, excessive sedentarism is a risk factor for psychological wellbeing in young adults. The amount of sedentary time—especially screen-based sitting—seems critical: even after accounting for exercise levels, high sedentary exposure remains significantly associated with greater psychological distress (18, 26). These data imply that meeting exercise recommendations alone is insufficient if the rest of the day is spent seated. To optimize mental health, interventions must also curb sedentary behavior through strategies such as frequent active breaks, reduced screen time, and greater incidental movement throughout the day.

### 4.2 The role of sleep and lifestyle synergies

A converging body of evidence indicates that the mental health benefits of physical activity are markedly amplified when adequate sleep is also achieved. Multiple analyses (13, 36) show that, although exercise independently confers protective effects, stress levels decline only when regular activity is paired with sufficient, high-quality sleep and minimal sedentary time (13). Sleep quality repeatedly emerges as a mediator linking physical activity to lower stress and depressive symptoms, a pattern that accords with the well-documented impact of sleep deprivation on emotion regulation, concentration, and mood. Consequently, interventions that integrate exercise promotion with sleep hygiene strategies and sedentary behavior reduction are likely to outperform single-behavior approaches in improving students' mental health.

### 4.3 Psychological mechanisms and theoretical contributions

The evidence base also illuminates the psychological pathways that link physical activity to better mental health. Numerous investigations have tested mediators such as psychological resilience, self-efficacy, life satisfaction, and academic burnout, indicating that exercise bolsters personal resources that buffer distress. For instance, one study reported that roughly half of the beneficial effect of physical activity on negative emotions was accounted for by increased resilience (23). Another found that higher life satisfaction and self-efficacy jointly mediated the relation between activity levels and reduced anxiety (32). Such findings align with frameworks—e.g., the Mastery Hypothesis and Self-Determination Theory—that view exercise as a context for competence, achievement, and social connection, thereby fostering positive affect and lowering emotional disorder risk. Physical activity may also act as a mild “innocuous stressor” that strengthens stress tolerance (4).

### 4.4 Positive mental health and academic implications

Our results align with earlier systematic reviews conducted in broader young-adult populations, which also reported inverse associations between physical activity and stress, anxiety, or depression. For example, Guerriero et al. concluded that sedentary lifestyles increased stress risk in university populations (3), while López-Valenciano et al. documented activity declines during the early COVID-19 period and their psychological consequences (10). However, by focusing specifically on studies published from 2020 onward and on university populations only, the present review extends previous evidence in three ways: (1) It demonstrates that perceived stress—often overlooked in earlier reviews centered mainly on anxiety and depression—is consistently lower among physically active students. (2) It integrates recent postpandemic data capturing fluctuations in lifestyle behaviors unique to this cohort. (3) It consolidates emerging research on mediators such as sleep quality, resilience, academic burnout, and problematic internet use, clarifying how and why physical activity confers psychological benefits.

Thus, evidence consistently shows that physical activity benefits both negative and positive facets of mental health. Overall, the current synthesis positions physical activity as a central behavioral determinant of student mental health. Although most included studies were cross-sectional, converging findings from quasi-experimental and longitudinal designs provide growing support for a prospective or causal relationship, especially when activity is combined with adequate sleep and reduced sedentary time. Concretely, active students not only present lower stress, anxiety, and depression scores but also report higher life satisfaction, positive affect, and overall psychological quality of life (28, 29). These patterns imply that regular exercise can promote flourishing and optimal functioning within the demanding university context, offering a readily accessible means to elevate mood and potentially enhance cognitive performance. In line with this, gains in executive functions—specifically inhibitory control—have been linked to physical activity, changes that could translate into better academic outcomes (14).

## 5 Limitations of the study

A critical examination of the included studies reveals several important considerations. To improve clarity, we streamlined the discussion of causal inference limitations and reinforced the role of methodological heterogeneity. Although most findings support a protective role of physical activity for stress and wellbeing, the majority of studies were cross-sectional, which restricts causal interpretation and leaves open the possibility of reverse associations (e.g., students with better mental health may simply be more inclined to engage in physical activity). Variations in measurement instruments, operational definitions of physical activity, and differences in stress or mental health scales may also have contributed to inconsistencies across studies. In addition, many investigations relied exclusively on self-reported tools such as the IPAQ or Pittsburgh Sleep Quality Index (PSQI), which are susceptible to recall and social desirability bias, while objective assessments (e.g., accelerometry, actigraphy, and physiological markers) remained scarce.

Contextual factors further limit comparability. Postpandemic lifestyle changes, fluctuations in academic workload, and institutional differences in campus life likely influenced both activity patterns and psychological outcomes. It is also worth noting that most samples originated from China, Portugal, or the United States, leaving relevant cultural contexts underrepresented. These limitations underscore the need for more diverse, methodologically robust, and context-sensitive research in future studies. The overrepresentation of women in many studies may have also influenced the observed associations, especially given the known gender differences in mental health symptoms. Despite these limitations, the convergent evidence suggests that interventions promoting both moderate-to-vigorous activity and reductions in sedentary time may offer meaningful benefits. Also, because most evidence is cross-sectional, definitive causal inferences are limited. Students who feel less depressed or stressed may simply be more inclined to exercise. The few longitudinal and experimental studies available mitigate this concern to some extent—for example, a semester-long follow-up showed that baseline physical activity preceded later reductions in anxiety and depression (43)—yet additional randomized controlled trials are still needed, especially those targeting perceived stress, which has received fewer intervention studies than anxiety or depression.

Publication bias is another possibility. The literature disproportionately features positive findings, so null or negative results may be underrepresented. Substantial heterogeneity in measurement tools, activity definitions, and cultural settings also complicates direct comparisons and meta-analysis. Even so, the narrative remains consistent across regions, lending support to a robust and generalizable link between physical activity and better mental health outcomes.

## 6 Future lines of research and practical implications

These findings strongly support the implementation of physical activity programs within universities as part of mental health promotion strategies. Universities could offer group exercise classes, active breaks in libraries, informative campaigns on the mind–body connection, or even curricular interventions, such as credit-based sports classes. Since academic workload is often cited as a barrier to exercise, institutions must provide convenient and appealing opportunities for students to stay active. Moreover, combining exercise promotion with resilience or stress management training may amplify benefits. For example, approaches such as “exercise + mindfulness” or “exercise + coping skills coaching” could simultaneously improve physical fitness and psychological resources.

Future research should aim to conduct randomized controlled trials with university populations, testing various exercise types (aerobic, strength, yoga, team sports) and assessing their impact on perceived stress, anxiety, depression, and academic outcomes. Further exploration is needed to determine the optimal intensity and dosage of physical activity, the effects in students with clinical disorders, and the potential drawbacks of excessive training. Although no study in this review reported about the negative effects of exercise, studying high-performance student athletes could clarify whether the relationship between activity and mental health remains linear or follows an inverted U-curve.

From an applied perspective, universities could adopt more concrete strategies such as integrating physical activity breaks into academic schedules, offering low-barrier group exercise programs, collaborating with sports departments to expand access to facilities, and incorporating physical activity into student support and counseling services. Embedding movement-based initiatives within existing wellbeing structures may enhance participation and provide scalable benefits for stress management and mental health promotion.

## 7 Conclusions

The evidence synthesized in this review consistently indicates that regular physical activity is associated with reductions in perceived stress and improvements in the mental health of university students. Across multiple contexts, physically active students reported lower levels of academic stress, depressive and anxious symptoms, as well as greater psychological wellbeing and better sleep quality compared with their sedentary peers. While most data originate from cross-sectional studies, findings from longitudinal and experimental research support a potential beneficial causal effect of exercise on mental health in university populations.

These results carry important practical implications. Higher education institutions should consider the promotion of physical activity as a core component of their psychological support and student wellbeing programs. Implementing policies that facilitate and encourage exercise (e.g., sports classes, proper infrastructure, and educational campaigns) will not only improve the physical health of students, but also help prevent mental health issues and mitigate academic stress, with potential collateral benefits for academic performance and retention. Moreover, comprehensive lifestyle interventions—combining physical activity, improved sleep hygiene, and reduced sedentary behavior—appear especially promising for maximizing positive effects on mental health.

In conclusion, physical activity emerges from this review as an accessible, low-cost strategy with multiple benefits to address the growing crisis of stress and mental health in university populations. Encouraging students to “move more and sit less” may be one of the most effective and sustainable ways to enhance their resilience, emotional wellbeing, and success during their academic journey. Universities, health professionals, and students themselves must recognize this powerful mind–body connection and work together to integrate regular exercise as part of a healthy university lifestyle.

## Data Availability

The original contributions presented in the study are included in the article/Supplementary Material; further inquiries can be directed to the corresponding authors.
